# Modular Nanotransporters Deliver Anti-Keap1 Monobody into Mouse Hepatocytes, Thereby Inhibiting Production of Reactive Oxygen Species

**DOI:** 10.3390/pharmaceutics16101345

**Published:** 2024-10-21

**Authors:** Yuri V. Khramtsov, Alexey V. Ulasov, Andrey A. Rosenkranz, Tatiana A. Slastnikova, Tatiana N. Lupanova, Georgii P. Georgiev, Alexander S. Sobolev

**Affiliations:** 1 Laboratory of Molecular Genetics of Intracellular Transport, Institute of Gene Biology of Russian Academy of Sciences, 34/5 Vavilov St., 119334 Moscow, Russia; ykhram2000@mail.ru (Y.V.K.); al.ulasov@gmail.com (A.V.U.); aar@genebiology.ru (A.A.R.); slacya@gmail.com (T.A.S.); tatyanalupanova@gmail.com (T.N.L.); georgiev@genebiology.ru (G.P.G.); 2 Faculty of Biology, Lomonosov Moscow State University, 1–12 Leninskie Gory St., 119234 Moscow, Russia

**Keywords:** modular nanotransporters, protein–protein interaction, Keap1, Nrf2, oxidative stress, CETSA

## Abstract

**Background/Objectives:** The study of oxidative stress in cells and ways to prevent it attract increasing attention. Antioxidant defense of cells can be activated by releasing the transcription factor Nrf2 from a complex with Keap1, its inhibitor protein. The aim of the work was to study the effect of the modular nanotransporter (MNT) carrying an R1 anti-Keap1 monobody (MNT_R1_) on cell homeostasis. **Methods:** The murine hepatocyte AML12 cells were used for the study. The interaction of fluorescently labeled MNT_R1_ with Keap1 fused to hrGFP was studied using the Fluorescence-Lifetime Imaging Microscopy–Förster Resonance Energy Transfer (FLIM-FRET) technique on living AML12 cells transfected with the *Keap1-hrGFP* gene. The release of Nrf2 from the complex with Keap1 and its levels in the cytoplasm and nuclei of the AML12 cells were examined using a cellular thermal shift assay (CETSA) and confocal laser scanning microscopy, respectively. The effect of MNT on the formation of reactive oxygen species was studied by flow cytometry using 6-carboxy-2′,7′-dichlorodihydrofluorescein diacetate. **Results:** MNT_R1_ is able to interact with Keap1 in the cytoplasm, leading to the release of Nrf2 from the complex with Keap1 and a rapid rise in Nrf2 levels both in the cytoplasm and nuclei, ultimately causing protection of cells from the action of hydrogen peroxide. The possibility of cleavage of the monobody in endosomes leads to an increase in the observed effects. **Conclusions:** These findings open up a new approach to specifically modulating the interaction of intracellular proteins, as demonstrated by the example of the Keap1-Nrf2 system.

## 1. Introduction

Recently, more and more attention has been paid to the study of oxidative stress in cells and the search for ways to prevent it. Nuclear factor erythroid 2-related factor 2 (transcription factor Nrf2) is one of the key components controlling the oxidative balance in cells [1,2,3]. Under normal conditions, Nrf2 forms a complex with its inhibitor, Kelch-like ECH-associated protein 1 (Keap1), which promotes Nrf2 destruction via the ubiquitin–proteasome system. The oxidation of Keap1, which contains numerous cysteine residues, decreases its affinity for Nrf2. As a result, the free Nrf2 enters the nucleus and activates a protective response via antioxidant response elements (AREs). The activation of the cell’s own antioxidant activity is considered to be a more effective way of protection from oxidative stress than the direct use of antioxidant compounds. It is one of the key approaches being studied for antioxidant therapy [4]. In addition to preventing oxidative stress, Nrf2 exerts anti-inflammatory effects and protects against environmental pollutants and poisons [5]. The protein–protein interaction of Nrf2-Keap1 is a promising drug target that is attracting increasing attention every year [6,7,8,9]. Numerous Nrf2 inducers imitate natural stress-induced Nrf2 activation by modifying Keap1 cysteines [10]. Such electrophilic compounds have a high probability of interacting with other cellular proteins, resulting in unwanted off-target effects [11,12]. This leads to the need for more specific approaches to Nrf2 activation, both in regard to intracellular regulation and in relation to tissue and cell types [5]. Using competitive inhibitors of Nrf2 and Keap1 interaction is another intracellular strategy to activate the Nrf2 pathway. In this context, a large number of peptides and small molecules are being investigated for potential applications [6,7,13,14]. This approach makes it possible to significantly increase specificity. Current estimates indicate that it is extremely difficult to generate an effective small molecule for the vast majority of known intracellular protein–protein interactions [15,16]. Peptides, antibodies, and antibody mimetics can provide a large area of contact with the targeted protein, resulting in increased specificity. This, in turn, reduces the probability of side effects. Furthermore, analysis of expression in the body shows that most intracellular proteins are specific to tissue or cell types [17]. The disadvantage of these relatively large compounds is their low cell penetration. In this context, a targeted intracellular delivery appears to be a viable option to affect these protein–protein interactions.

Affecting the Keap1/Nfr2/ARE system is considered a way to treat a number of pathologies caused by oxidative stress, including neurodegenerative and cardiovascular diseases, cancer, diabetes mellitus, inflammation and intoxication [8,18,19]. An example of the latter is protection against liver damage caused by oxidative stress when exposed to high doses of acetaminophen [20]. In addition, for a number of viruses, including SARS-CoV-2, the antiviral activity of drugs that activate the Keap1/Nfr2/ARE system has been discovered [5].

Selective activation of the Nrf2 system can be provided by competition between its Keap1 inhibitor and corresponding polypeptide sequences. The difficulty in delivering active drugs that do not penetrate cells can be overcome by employing modular nanotransporters (MNTs). An MNT is a technological platform intended to deliver active principles into the specified compartment of target cells [21]. MNTs consist of several modules, each with its own function. A ligand module is used to bind to internalizable receptors on the surface of target cells. An endosomolytic module is used to exit closed compartments, endosomes, into the cytosol. An effector module is used to deliver MNTs to the desired cellular compartment or to the desired target protein. The optimal spatial integration of all modules is achieved by a carrier module, which also imparts the necessary solubility to the MNTs. The carrier module is the largest module and is often used to attach molecules delivered to the cell, such as photosensitizers or radioisotopes. Originally designed to deliver anticancer drugs into the nucleus of tumor target cells, MNTs have demonstrated significant potential and can be used to target intracellular protein–protein interactions. The use of MNT technology can provide a more targeted effect on intracellular protein–protein interactions via the high-affinity binding of amino acid sequences and delivery to specified tissues or cells. Our studies have shown the feasibility of this approach for several intracellular protein targets, for example, for the SARS-CoV2 N-protein [22].

Recently, we have also developed MNTs that contain an anti-Keap1 monobody and are able to interact with Keap1 in the cytosol of target cells [23], but the consequences of this interaction for cell homeostasis remain unknown. Therefore, the verification of the effect of these MNTs on Nrf2-Keap1 interactions, representing the first step of the antioxidant response, is of great interest. Here we present the results of evaluating the effectiveness of the recently developed MNT in blocking the interaction between Nrf2 and Keap1 in cultured cells, as well as its consequences for protecting cells from the effects of hydrogen peroxide. In addition, a change in the efficiency of the MNT, with the possibility of cleaving off its part interacting with Keap1 in endosomes, was shown. Mouse hepatocytes were chosen as a cell model, since oxidative stress caused by liver intoxication is one of the most common phenomena in the world.

## 2. Materials and Methods

### 2.1. Cell Lines

The murine hepatocyte AML12 and human embryonal kidney HEK293T cells were obtained from the American Type Culture Collection (ATCC, Manassas, VA, USA). The AML12 cells were maintained in DMEM/F12 medium (PanEco, Moscow, Russia) supplemented with 10% fetal bovine serum, 10 μg/mL insulin, 5.5 μg/mL transferrin, 5 ng/mL selenium, 40 ng/mL dexamethasone, and 50 μg/mL gentamycin according to ATCC guidelines. The HEK293T cells were cultivated in DMEM/F12 medium (PanEco) with 10% fetal bovine serum and 50 μg/mL gentamycin.

### 2.2. Activation of AREs with Keap1-Binding Modules on the MNT Scaffold

To find the best MNT variant capable of interacting with Keap1 and disrupting its binding to Nrf2, we used a gene that contained sequences from previously used MNT modules [21]. The diphtheria toxin translocation domain (DTox) sequence was employed as an endosomolytic module. The sequence of the hemoglobin-like HMP protein was taken as a carrier module. The optimized nuclear localization signal (NLS) of the large T-antigen of the SV40 virus was used as a module for delivery into the cell nucleus. The ligand module of the MNT was replaced by a yellow fluorescent protein, TurboYfp. For the initial testing of different Nrf2/ARE system-activating modules as a base vector, we used the *TurboYfp-DTox-HMP-NLS* plasmid, which had been generated by subcloning *DTox-HMP-NLS* modules to the *pTurboYfp-C* plasmid (Evrogen, Moscow, Russia). Next, we subcloned different amino acid sequences to the C-end of the TurboYfp-DTox-HMP-NLS protein to compare their relative efficiency in activating the Nrf2/ARE system after transfection. As such, we tested fragments of Keap1-binding motifs of different lengths from Nrf2, p62 [24], prothymosin α [25], and the full-sized p21 protein [26], as well as an artificial antibody-like protein anti-Keap1 monobody [27]. The amino acid sequences of fragments from Nrf2, p62, and prothymosin α are shown in Table 1. A DNA fragment encoding the anti-Keap1 monobody (R1) was synthesized by General Biosystems (Morrisville, NC, USA) based on a published amino acid sequence [27]. Constructs lacking the NLS module were produced with the QuickChange^TM^ site-directed mutagenesis kit (Agilent Technologies, Santa Clara, CA, USA).

To evaluate the functionality of different modules activating the Nrf2/ARE system, a cell model was developed based on the HEK293T cells co-transfected with several plasmids: a target plasmid expressing the MNT fused with TurboYfp (to assess transfection efficiency), and the plasmids *pGL4.37* and *pRL-Tk* (both Promega, Madison, WI, USA). *pGL4.37* expresses firefly luciferase under the control of a minimal promoter and several AREs. *pRL-Tk* expresses Renilla luciferase under the control of a constitutive promoter and serves to normalize the signal received from *pGL4.37*. Transfection of HEK293T cells was performed using polyplexes, as previously described [28], in triplicates for each experiment [28]. For transfection, the plasmids were used in a ratio of 15:3.5:1.5 (MNT plasmid, *pGL4.37*, and *pRL-Tk* plasmids, respectively). Luciferase activity was measured 48 h after transfection using a GloMax luminometer (Promega) and a dual luciferase assay kit (Promega), following the manufacturer’s protocol.

### 2.3. Modular Nanotransporters (MNTs)

After initial testing, constructs lacking NLS with and without the R1 monobody were subcloned into the *(affibody_EGFR_)-DTox-HMP-NLS* plasmid. The plasmids encoding MNTs were verified by sequencing and retransformed in *E. coli* BL21 (DE3) cells. MNTs with the R1 monobody against Keap1 (MNT_R1_) as an effector module and MNTs lacking the monobody (MNT_0_) both contained the Z1907 affibody as a ligand module for the MNTs binding to the epidermal growth factor receptor (EGFR) and receptor-mediated endocytosis, the translocation domain of diphtheria toxin as an endosomolytic module for endosome escape, and the *E. coli* HMP hemoglobin protein as a carrier module that combines all modules together. Using the QuickChange^TM^ site-directed mutagenesis kit (Agilent Technologies, Santa Clara, CA, USA), an MNT containing the FKFL cleavage site [22] for the detachment of the R1 monobody in endosomes was generated. This MNT is hereinafter designated as MNT_clR1_. All MNTs were produced in *E. coli* BL21(DE3) strains and purified as described previously [23]. Briefly, to express MNTs in *E. coli*, 0.5 mM of isopropyl-β-d-1-thiogalactopyranoside was added overnight at 18 °C. The bacterial biomasses were lysed in an ice-cold solution containing 50 mM sodium phosphate, pH 8.0, 300 mM sodium chloride, 10 mg/mL lysozyme, 1 mM phenylmethylsulfonyl fluoride, and 0.5% Triton X-100. Both MNTs were purified using Ni-Sepharose 6 Fast Flow prepacked columns (Cytiva, Marlborough, MA, USA) according to the manufacturer’s instructions, to a purity above 95%. Finally, the MNTs were dialyzed against phosphate-buffered saline (10 mM sodium phosphate, 150 mM sodium chloride, pH 7.4), sterilized by filtration and freeze-dried.

### 2.4. Confocal Laser Scanning Microscopy (CLSM)

Immunofluorescence was used to examine the ability of MNTs to activate the Nrf2 signaling pathway in cells. To do this, AML12 cells were seeded on coverslips in the wells of 24-well culture plates. Two days later, the medium was replaced with a fresh one, and either MNT_R1_ or MNT_0_ at a final concentration of 500 nM was added to the cells. The cells were then incubated in a CO_2_ incubator for the prescribed time. Sulforaphane (LKT Labs, St. Paul, MN, USA) added to a final concentration of 10 μM for 2 h served as a positive control for Nrf2 activation. At the end of the incubation time, the cells were washed three times with Hanks’ solution and fixed with ice-cold methanol for 15 min at −40 °C. Fixed cells were incubated in blocking solution (5% non-fat milk, 1% bovine serum albumin, and 0.1% Tween-20 in PBS) for 35 min, followed by incubation with an anti-Nrf2 antibody (ab31163, Abcam, Waltham, MA, USA) diluted to 1:150 in blocking solution overnight at 4 °C. Then the cells were washed 3 times with PBS containing 0.1% Tween-20, incubated with Alexa FluorTM 555 labeled Goat anti-Rabbit IgG (H + L) Cross-Adsorbed Secondary Antibody, (A-21428, Invitrogen, Carlsbad, CA, USA) for 1 h at 37 °C, and washed 3 times with PBS containing 0.1% Tween-20 again. Afterwards, nuclei were counterstained with a 1 μg/mL solution of 4′,6-diamidino-2-phenylindole (DAPI); the cells were rinsed with PBS and mounted on microscope slides with Immu-Mount (Thermo Fisher Scientific, Waltham, MA, USA, #9990402). CLSM images were acquired using a STELLARIS 5 fluorescence confocal microscope (Leica Microsystems, Wetzlar, Germany) with a ×63 lens, NA 1.4 at 0.87 Airy units, using 405 nm and 561 nm laser lines. The cytoplasmic and nuclear fluorescent signals attributed to the Nrf2 protein were quantified using a modified version of the Cyt/Nuc macro for Image J [29]. In brief, the DAPI-stained nuclei from the DAPI fluorescent channel were identified and further applied as a mask to the Alexa FluorTM 555 channel corresponding to Nrf2 distribution. This way, Nrf2-related fluorescence in the nuclei was measured. The fluorescence in the Alexa FluorTM 555 channel that was not colocalized with DAPI fluorescence was regarded as a cytoplasmic Nrf2-related. The obtained fluorescent signal for the nuclei and the cytoplasmic fluorescence for the cells incubated with MNTs was further normalized to that of the non-treated control cells. For each point, at least 10 fields of view with more than 170 cells were analyzed. All statistics were calculated using the GraphPad Prism 6 software package (GraphPad Software Inc., La Jolla, CA, USA). The autofluorescence of cells processed omitting all antibodies and the non-specific fluorescent signals of cells that were stained, omitting only anti-Nrf2 antibodies, were also recorded, and their contribution to the resulting fluorescent signal was taken into account.

### 2.5. The Cellular Thermal Shift Assay (CETSA)

The cellular thermal shift assay (CETSA) [30,31] was carried out according to the following protocol. Cells from a 25 cm^2^ culture flask were trypsinized and pelleted. The resulting cell pellets were suspended in a pH 8.0 buffer solution that contained 1.5 μg/mL aprotinin, 150 mM sodium chloride, 5 mM EDTA, 0.174 mg/mL phenylmethylsulfonyl fluoride, and 25 mM sodium phosphate. A MACSQuant Analyzer flow cytometer (Miltenyi Biotec GmbH, Paris, France) was used to count the cells. Nrf2 melting curves were obtained from both intact cells and cell lysates as described previously [32]. In experiments to quantify the concentration of MNT_R1_ in cells, cell lysis was performed in an analogous way, namely by four cycles of freezing in liquid nitrogen and thawing at 37 °C. The cell lysates were then centrifuged at 8000× *g* for 60 min at 4 °C in both cases. The resulting supernatants were loaded onto a 10% SDS-PAGE gel and then Western blotted with anti-Nrf2 antibody (ab31163, Abcam, Cambridge, UK), used for these purposes previously [32]. The Trans-Blot Turbo Transfer System (Bio-Rad, Hercules, CA, USA) was used to transfer proteins from gels to 0.22 μm supported nitrocellulose membranes. The signal intensity was measured in band areas as well as background areas. Thus, the measured band intensity was averaged over three replicates loaded on gel and normalized to the band intensity of the sample, in which the heating step was excluded. The resulting curve was fitted by the logistic sigmoid function using Origin 6.0 software (OriginLab Corporation, Northampton, MA, USA).

### 2.6. Estimating the Intracellular Concentration of MNT_R1_

AML12 cells were incubated with 500 nM MNT_R1_ for 5, 10 or 15 min. Next, the cells were washed with Versene solution, trypsinized and pelleted by centrifugation. The resulting cell pellets were suspended in a pH 8.0 buffer solution that contained 1.5 μg/mL aprotinin, 150 mM sodium chloride, 5 mM EDTA, 0.174 mg/mL phenylmethylsulfonyl fluoride, and 25 mM sodium phosphate. A MACSQuant Analyzer flow cytometer (Miltenyi Biotec GmbH, Paris, France) was used to count the cells. Cells were lysed with four cycles of freezing in liquid nitrogen and thawing at 37 °C. The cell lysates were then centrifuged at 8000× *g* for 30 min at 4 °C. The resulting supernatants were loaded onto a 10% SDS-PAGE gel and then Western blotted with anti-MNT antibody (rabbit polyclonal antibodies to MNT containing HMP and the translocation domain of diphtheria toxin, ALMABION, Voronezh, Russia). Samples with a known concentration of MNT_R1_ were also loaded onto the gel. The Trans-Blot Turbo Transfer System (Bio-Rad, Hercules, CA, USA) was used to transfer proteins from gels to 0.22 μm supported nitrocellulose membranes. The signal intensity was measured in band areas as well as background areas. Thus, the measured band intensity was averaged over several replicates loaded on gel, and the MNT_R1_ concentrations in cell lysates were calculated by a linear interpolation of samples with known concentrations. The concentration of MNT_R1_ in the cytoplasm of cells was calculated based on the concentration of cells, the volume of their cytoplasm and nuclei and the concentration of MNT_R1_ in cell lysates.

### 2.7. Thermophoresis

The interaction affinities between MNT_R1_ or cleaved MNT_clR1_ and tKeap1 (the Keap1 C-terminal fragment (aa 312–624), responsible for the Nrf2 binding) were measured with a Monolith NT.115 instrument (NanoTemper Technologies, München, Germany) in phosphate buffer (25 mM NaH_2_PO_4_ (Sigma-Aldrich, Taufkirchen, Germany), 150 mM NaCl, pH 8.0). The tKeap1 protein was labeled with ATTO647 fluorescent dye. To do this, a 10-fold molar excess of the activated ATTO647-N-hydroxysuccinimide ester (Lumiprobe, Moscow, Russia) was added to the tKeap1 protein in 65 mM carbonate buffer (pH 8.5), and the mixture was incubated for 1 h at room temperature with constant stirring. The tKeap1 protein with attached ATTO647 was separated from the free dye using a PD10 chromatographic column. As a result, on average, two ATTO647 molecules were attached to one tKeap1 molecule. At a fixed concentration of tKeap1-ATTO647 (0.12 or 5 nM), thermophoresis curves were obtained. Four such curves were obtained for each experiment, and the whole experiment was repeated four–five times. For each curve, the dissociation constants of the tKeap1 complex with MNT_R1_ or cleaved MNT_clR1_ were determined by MO.Affinity Analysis v2.3 (NanoTemper Technologies, München, Germany); they were averaged over all curves, and the relative measurement errors were determined. Cleaved MNT_clR1_ was prepared by incubating 4 μM MNT_clR1_ with 4 μg/mL activated native human cathepsin B (ab90387, Abcam, Cambridge, UK) for 20 h at 37 °C. The activation of cathepsin B was carried out as described by Kern et al. [33].

### 2.8. FLIM-FRET Analysis of Intracellular MNT_R1_-Keap1 Interactions

Fluorescently labeled MNT_R1_ and hrGFP-Keap1, produced in AML12 cells transfected with Addgene plasmid *# 28025*, bearing the *hrGFP-Keap1* gene, were used for the analysis. MNT_R1_ was labeled with the N-hydroxysuccinimide ester of the fluorescent dye AF568 (Lumiprobe, Moscow, Russia) as previously described to investigate its interaction with Keap1 in the cell [23]. The transfection of AML12 cells was accomplished according to the instructions of the K2^®^ Transfection System (Biontex Laboratories GmbH, Martinsried, Germany) as reported earlier [23]. The interaction between MNT_R1_ and Keap1 was demonstrated using fluorescence lifetime-based fluorescence resonance energy transfer (FLIM-FRET) in the cell [34,35]. Transfected AML12 cells were incubated with 500 nM of fluorescently labeled MNT_R1_ for the indicated time at 37 °C in serum-free medium. After that, the cells were washed with Hanks’ solution for FLIM-FRET experiments with an LSM-510 META NLO multiphoton confocal laser scanning microscope (Carl Zeiss, Oberkochen, Germany) with a 63× lens (NA 1.4). The microscope was equipped with a Mai Tai Broadband femtosecond laser (SpectraPhysics, Irvine, CA, USA) and a time-correlated single-photon counting system (TCSPC from Becker and Hickl GmbH, Berlin, Germany). A femtosecond laser was used to generate hrGFP fluorescence at 800 nm via two-photon excitation. hrGFP fluorescence was captured at wavelengths ranging from 500 to 550 nm. In the Origin6.0 program, frequency distributions of the mean fluorescence lifetimes of hrGFP, τ_m_, were plotted for the cells to which MNT was not added or which were incubated with MNT for a specified time. These dependencies were fitted in the same program using Gaussian curves. The number of Gaussian curves was chosen to be minimal, at which the determination coefficient, r^2^, became higher than 0.98.

### 2.9. Intracellular Reactive Oxygen Species Assay

The generation of ROS was detected using 6-carboxy-2′,7′-dichlorodihydrofluorescein diacetate (cDCFH-DA, Lumiprobe, Moscow, Russia). cDCFH-DA does not produce fluorescence, but it can enter the cells. It is deacetylated in the cell and can transform into a fluorescent form, carboxy-2′,7′-dichlorofluorescein (cDCF), when exposed to ROS. For these experiments, AML12 cells (15–25 cell passage) were seeded into 48-well plates of 75,000 cells per well. The next day, the medium in the wells was replaced with DMEM/F-12 medium without serum (PanEco), either with or without the necessary MNT additions. After a certain incubation time (5, 10 or 15 min) in a CO_2_ incubator (37 °C, humidity 94%), the medium was withdrawn from the wells, and a fresh medium without serum was added for the indicated time. Before the measurement, the medium was changed to a medium without serum containing 100 μM cDCFH-DA. After 30 min of incubation with cDCFH-DA, the cells were washed with PBS containing sodium and potassium chloride, followed by the addition of a freshly prepared 10 mM hydrogen peroxide solution in PBS. The cells were washed and trypsinized after being incubated with peroxide in the dark for 15 min at room temperature. The resulting cell suspensions were centrifuged and diluted in Hanks’ solution. A CytoFLEX S (Beckman Coulter, CA, USA) flow cytometer was used to quantify cDCF fluorescence in the cells. cDCF fluorescence was detected only in living cells, which were determined by the absence of fluorescence in the propidium iodide channel. The results are presented as the difference between the fluorescence of peroxide-treated cells and the fluorescence of untreated control cells without any additions. Using GraphPad Prism 6, the obtained data were tested for a normal distribution and, in the case of normal distribution, Dunnett’s multiple comparisons test was used to assess the statistical significance of the differences. In the case of a non-normal distribution, Dunn’s multiple comparisons test was used.

## 3. Results

### 3.1. Selection of Modules for Interaction with Keap1

We examined a variety of modules with relevant protein sequences to develop an MNT capable of interacting with Keap1 within cells efficiently. To achieve this, genetic constructs containing the endosomolytic, carrier, and nuclear localization modules (an MNT scaffold performing transport functions) were fused to the N-terminus of the examined modules. The ligand module was replaced by the TurboYFP sequence in the genetic construct for eukaryotic expression. Such an experimental setting allowed us to fix parameters that can be potentially affected by receptor-mediated endocytosis and the efficiency of the subsequent intracellular transport of new MNTs and perform a screening of MNT-(Nrf2 fragment) variants. HEK293T cell transfection with reporter plasmid pGL4.37-encoding firefly luciferase under the control of four copies of AREs has been explored successfully as a test system to assess the ability of the developed constructs to activate the Nrf2/ARE signaling pathway (Table 1). The short hydrophilic spacer RSHD between the MNT scaffold and the module with the short sequence from Nrf2 (LQLDEETGE) increased its activity by 3.0 times (*p* < 0.005, Mann–Whitney test). We investigated sequences of different lengths with an ETGE motif (constructs 1–3), which is a necessary part of the sequence for the high-affinity binding of Nrf2 to Keap1, DEETGE [36,37,38,39]. To optimize the most efficient, construct 3, we implemented a mutant MNT scaffold with deleted NLS (construct 4) to investigate the relevance of the interaction with nuclear Keap1, as Keap1 is localized both in the nucleus and in the cytoplasm. Indeed, construct 4 with deleted NLS was more efficient than the nucleus-targeted one, which is consistent with the higher Keap1 cytoplasmic level [40]. By the same token, we took advantage of the anti-Keap1 R1 monobody, which has a higher affinity for Keap1 than Nrf2 [27]. We obtained the R1 monobody via gene synthesis and fused it to the C-end of the MNT scaffolds with and without NLS (constructs 5–6). As with the sequence from natural Nrf2, the construct without NLS was revealed to be more successful when interacting with Nrf2 in the cytoplasm. The efficiency of the cytoplasm-directed R1 construct 6 was a bit higher than for the cytoplasm-directed construct fused with the long ETGE motif (construct 4).

In addition to the obvious selection of Nrf2 fragments and antibody mimetics, we have studied Keap1-binding motifs from cellular proteins competing with Nrf2, such as p62 [41] and prothymosin α [25]. However, we did not observe a great activation of luciferase expression under ARE control, which could be attributed to the low affinity of these proteins’ motifs for Keap1 (Table 1). Another potential variant of the Nrf2 system activator, p21 [26], did not show a better enhancement of luciferase activity, either when fused to TurboYfp or in a free state. The NLS-deprived constructs with monobody R1 and the longest NRF2 sequence (constructs 4 and 6) showed the most promise for ARE activation in this cell system. The fluorescent protein was replaced with an affibody against EGFR in these genes for subsequent experiments, and MNTs were produced in *E. coli* BL21(DE3) as described in the Section 2. MNT_R1_ had a substantially better yield than MNT containing the NRf2 sequence; therefore, it was employed in subsequent experiments.

### 3.2. Effect of MNTs on the Level of Nrf2 in AML12 Cells

**Figure 1:**
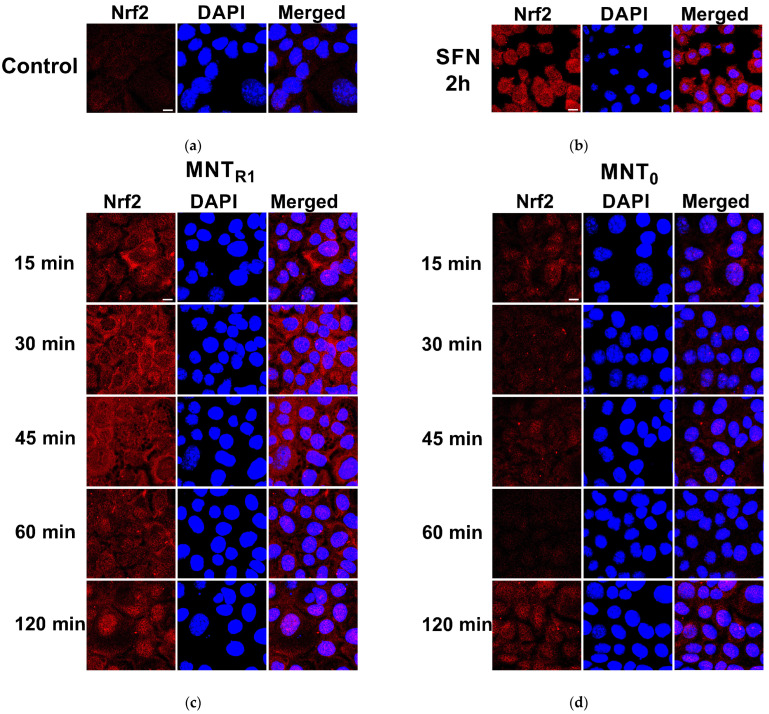
The changes in the Nrf2 level in AML12 cells following MNT or sulforaphane addition. MNT_R1_ or MNT_0_ were added to AML12 cells for the indicated time. The fixed cells were stained by indirect immunofluorescence. Nrf2 was revealed by immunofluorescence (red); cell nuclei were stained with DAPI (blue). (**a**) Representative images of cells without any MNT addition (no additives); (**b**) cells after 2 h incubation with 10 µM of sulforaphane; (**c**,**d**) cells after incubation for indicated time with 500 nM of MNT_R1_ and MNT_0_, respectively. Bar—10 µm.

The properties of all the modules of MNT_R1_ were described in a previous publication [23]. MNT_R1_ is able to bind to EGFR with a dissociation constant of 63 nM and provide the destruction of the phospholipid bilayer at pH 5.5, corresponding to the pH of the endosomes. We also discovered that MNT_R1_ has a high affinity for Keap1 (the equilibrium dissociation constant was 5.4 ± 0.5 nM; see also Supplementary Figure S1), in contrast to MNT_0_, which has a much lower affinity for Keap1 (93 nM) [23]. To see how these differences would alter the level and distribution of Nrf2 in AML12 cells, we performed the CLSM imaging of samples at various time points following the MNT addition. Sulforaphane, a potent Nrf2-activating agent, significantly enhanced a fluorescent signal from immunofluorescently detected Nrf2 both in the cytoplasm and in the nuclei after 2 h of incubation (Figure 1a,b). In comparison to control cells, Nrf2 fluorescence increased by 3.7 ± 0.2 in the nucleus and 3.5 ± 0.1 in the cytoplasm (mean ± SE, *n* = 10). This indicates a disturbance of the binding of Nrf2 to Keap1 and a decrease in its degradation. It should be noted that under these conditions, sulforaphane caused a decrease in the number of AML12 cells and a change in their morphology (Figure S2).

**Figure 2:**
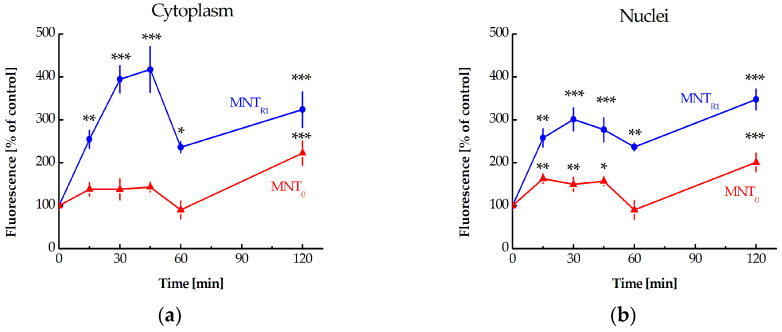
Kinetics of changing Nrf2 levels after the addition of MNT_R1_ and MNT_0_ for the cytoplasm (**a**) and nuclei (**b**), respectively. Data are presented as mean ± SE. * *p* < 0.05, ** *p* < 0.01, *** *p* < 0.001.

The addition of 500 nM of MNT_R1_ to AML12 cells resulted in a fast increase in the fluorescent signal from Nrf2 (Figure 1c). It should be noted that the concentrations of MNT_R1_ used at the selected times of their incubation did not result in toxic effects or morphological changes in cells (Figure S2). The data quantification demonstrates that MNT_R1_ was significantly more effective in impacting on Nrf2 levels both for the cytoplasm and nuclei for all investigated time points than MNT_0_ (Figure 1d). The response of cells to the addition of MNT was quite rapid: already at 15 min after the addition of MNT_R1_, the increase in fluorescence in the nucleus and cytoplasm reached 2.6 and 2.5 folds, respectively. After a rapid increase for the first 45 min of incubation, the level of Nrf2-attributed fluorescence decreased at one hour and then began to increase again by the second hour of incubation (Figure 2). The level of Nrf2 detected at the end of the two-hour incubation with MNT_R1_ was similar to that caused by sulforaphane. By this time, the level of Nrf2 had increased 3.5 ± 0.2 times in the nuclei and 3.2 ± 0.4 times in the cytoplasm (mean ± SE, *n* = 15). Unlike sulforaphane, MNT_R1_ did not affect the shape of AML12 cells.

The rapid increase in the level of Nrf2 in cells to which MNT_R1_ was added (Figure 2) raises the question of the mechanism of changing the level of Nrf2. To clarify this issue, we investigated the processes occurring in AML12 cells early after the addition of MNT_R1_.

### 3.3. Studying Interaction Between MNT_R1_ and Keap1 by FLIM-FRET

**Figure 3:**
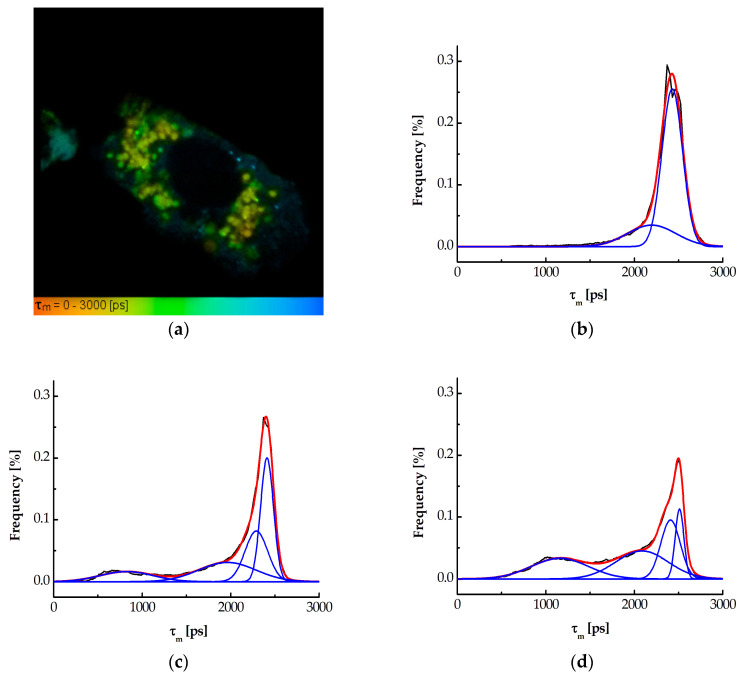
Analysis of the interaction between intracellular Keap1-hrGFP and MNT_R1_-AF568 after its addition to AML12 cells with the temporary expression of Keap1-hrGFP. Imaging mean fluorescence lifetimes of hrGFP, τ_m_, in the cell after one hour of incubation with 500 nM MNT_R1_-AF568 (**a**). Frequency distributions of mean fluorescence lifetimes of hrGFP, τ_m_, in cells that were not treated with MNT_R1_-AF568 (**b**), incubated for 15 min with 500 nM MNT_R1_-AF568 (**c**), and incubated for one hour with 500 nM MNT_R1_-AF568 (**d**). The curves were averaged over 5 to 15 cells. The black lines represent the average curves; the blue lines are a result of their fitting with Gaussian curves; and the red lines show the summation of the Gaussian curves.

Previously [23], we demonstrated that MNT_R1_ can interact with the Keap1 protein using the thermophoresis method (see also Supplementary Figure S1). Using the FLIM-FRET technique, we have also shown that this interaction also occurs inside the cell [23]. Now we have tried to estimate the proportion of Keap1 molecules in the cell with which MNT_R1_ molecules entering the cell can interact. To do this, we used the same approach as before. AML12 cells temporally expressing the Keap1 protein fused with the fluorescent hrGFP protein were produced. MNT_R1_ was labeled with the fluorescent dye AF568, enabling resonance energy transfer via the Förster mechanism. FRET detection can be performed via the mean hrGFP fluorescence lifetime, τ_m_, which will decrease if the acceptor is available. Indeed, the FRET between hrEGFP and AF568 was detected as was previously shown. This indicated the formation of a complex between MNT_R1_ and the Keap1 protein. Keap1 fused to hrGFP was not uniformly distributed in the cell and had a characteristic mean fluorescence lifetime of about 2400 ps (Figure 3a, green regions). In the case of complex formation, the τ_m_ of hrGFP was noticeably reduced to about 1000 ps (Figure 3a, yellow regions). We analyzed the contribution of fluorophores with a different τ_m_ to the observed fluorescence decay kinetics. Figure 3b–d show the frequency distributions of hrGFP τ_m_ averaged over a variety of cells for control AML12 cells without MNT_R1_ and AML12 cells incubated with 500 nM MNT_R1_-AF568 for 15 min and 1 h, respectively.

These data indicate that the rapid increase in the level of Nrf2 in cells may be caused by the interaction between MNT_R1_ and Keap1. To verify that this interaction can quickly affect the state of Npf2, we used CETSA.

### 3.4. Studying Interaction Between MNT_R1_ and Keap1 by CETSA

**Figure 4:**
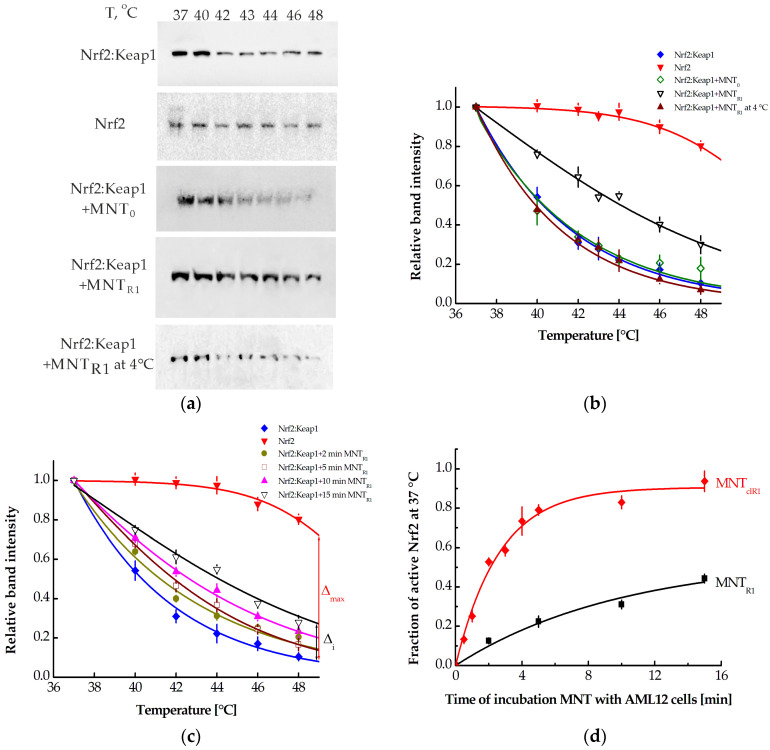
Studying the effect of MNT on the Nrf2 microenvironment by CETSA. (**a**) Examples of immunoblots of Nrf2 in complex with Keap1 (cell heating), active Nrf2 (the cell lysate with MNT_R1_ heating), after 15 min of incubation of AML12 cells with 500 nM MNT_R1_ (cell heating), after 15 min of incubation of AML12 cells with 500 nM MNT_0_ (cell heating), and after 15 min of incubation of AML12 cells with 500 nM of MNT_R1_ at 4 °C (cell heating). (**b**) Melting curves of Nrf2 in complex with Keap1 (blue curve), active Nrf2 (red curve), after 15 min of incubation of AML12 cells with 500 nM MNT_R1_ (black curve), after 15 min of incubation of AML12 cells with 500 nM MNT_0_ (green curve), and after 15 min of incubation of AML12 cells with 500 nM of MNT_R1_ at 4 °C (brown curve). The data were obtained by a CETSA using immunoblotting with antibodies against Nrf2. The dependences are normalized to the average intensity of the band corresponding to Nrf2 at 37 °C. (**c**) Melting curves of Nrf2 in complex with Keap1 (blue curve), active Nrf2 (red curve), and incubation of AML12 cells with 500 nM MNT_R1_ for 2 min (dark yellow curve), 5 min (wine curve), 10 min (magenta curve), and 15 min (black curve). The data were obtained by a CETSA assay using immunoblotting with antibodies against Nrf2. The dependences are normalized to the average intensity of the band corresponding to Nrf2 at 37 °C. (**d**) The dependence of the fraction of active Nrf2 at 37 °C on the incubation time of AML12 cells with 500 nM of MNT_R1_ (black curve) or MNT_clR1_ (red curve). The average values of ± standard error (n = 4–14) are provided.

The Nrf2 system’s activation starts with the disruption of the Nrf2:Keap1 complex, which normally contains the majority of Nrf2 molecules in cells. Section 3.1 demonstrates a rapid increase in the level of Nrf2 in the AML12 cells in response to the addition of MNT_R1_. To identify changes in the Nrf2 microenvironment, we examined its thermal stability using CETSA based on immunoblotting data with antibodies against Nrf2. The melting curves of Nrf2 in cells were obtained by heating cell samples to different temperatures within three minutes, followed by cell lysis, centrifugation, and Western blot analysis of the supernatant (Figure 4a; a typical example of a complete Western blot is shown in Figure S3). In AML12 cells incubated without MNT_R1_, Nrf2 was in complex with Keap1, as indicated by the blue melting curve in Figure 4b. The red curve corresponds to the melting curve of active Nrf2 with a disrupted Nrf2-Keap1 interaction, obtained from cell lysate incubated with 1 μM of MNT_R1_. The disruption of the Nrf2:Keap1 complex significantly shifts the Nrf2 melting curve to the high temperature range. When AML12 cells were treated with 500 nM MNT_R1_ for 15 min, the melting curve of Nrf2 (Figure 4a; Figure 4b, black curve) shifted significantly towards the melting curve of active Nrf2 (Figure 4a; Figure 4b, red curve). The addition of MNT_0_ to AML12 cells did not alter the melting curve of Nrf2 (Figure 4a; Figure 4b, green curve). Incubation with MNT_R1_ at 4 °C did not affect the melting curve of Nrf2 (see Figure 4a; Figure 4b, the brown curve). These results demonstrate that the increase in the Nrf2 level in AML12 cells following incubation with MNT_R1_ (Figure 2) was due to MNT_R1_ interaction with Keap1.

Similar melting curves were obtained by incubating AML12 cells with MNT_R1_ for 2, 5, 10, and 15 min and subsequent washing (Figure 4c). Using the obtained melting curves, it is possible to determine the proportion of active Nrf2 in the cell, as we described earlier [32]. To do this, we need to know the relative distance (Δ_i_) between the melting curves of Nrf2 at a temperature (t_i_) in control cells and in cells that were incubated with MNT_R1_ for the specified time (Figure 4c). Then, at the same temperature, the distance between the melting curves of active Nrf2 and Nrf2 in complex with Keap, Δ_max_ (Figure 4c), is calculated. The fraction of active Nrf2 at a given temperature is F(t_i_) = Δ_i_/Δ_max_. It turned out that in the temperature range of 40–44 °C, this proportion does not depend on temperature. For example, at 15 min of incubation with MNT_R1_, this proportion is 0.45 ± 0.04, 0.45 ± 0.05, and 0.43 ± 0.03 at temperatures of 40, 42 and 44 °C, respectively. Therefore, it can be assumed that at physiological temperatures, i.e., at 37 °C, the fraction of F will be the same as at these temperatures, and this fraction was estimated by averaging the corresponding values of F(t_i_) in the temperature range of 40–44 °C. The dependence of this proportion on the incubation time of AML12 cells with MNT_R1_ is shown in Figure 4d (black curve).

MNT_R1_ concentrations in AML12 cells were also estimated after 5, 10, and 15 min of incubation with MNT_R1_. The dilution coefficient after cell lysis was calculated for each sample using flow cytofluorometry to determine the concentration of AML12 cells as well as the volumes of cells and nuclei [32]. A Western blot with antibodies against MNT was performed for cell lysates (Figure S4). As a control, mixtures of AML12 cell lysate, to which MNT was not added, with solutions with known concentrations of MNT_R1_ were used. Calibration curves were used to determine MNT_R1_ levels in cell lysates and then in the cytoplasm using a dilution factor. The concentration of MNT_R1_ was calculated by considering both full-size molecules and high-molecular-weight catabolites (Figure S4). It was estimated that the concentration of MNT_R1_ in AML12 cells was 264 ± 30, 286 ± 21, and 420 ± 47 nM for the incubation time points of 5, 10, and 15 min, respectively.

### 3.5. Protecting AML12 Cells Against Hydrogen Peroxide

**Figure 5:**
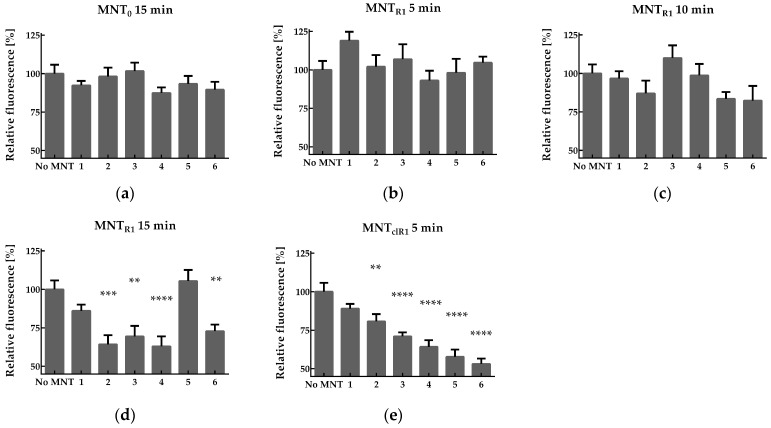
Effect of MNTs on ROS generation. The effect of pre-incubating AML12 cells with 500 nM MNT_0_ for 15 min on cDCF fluorescence at different time points (1–6 h after adding MNT_0_) is shown in plot (**a**). The effect of pre-incubating AML12 cells with 500 nM MNT_R1_ for 5, 10, and 15 min on cDCF fluorescence at different time points (1–6 h after adding MNT_R1_) is shown in plots (**b**), (**c**), and (**d**), respectively. The effect of pre-incubating AML12 cells with 500 nM MNT_clR1_ for 5 min on cDCF fluorescence at different time points (1–6 h after adding MNT_clR1_) is shown in plot (**e**). Data are presented as mean ± SE (*n* = 6–18). The significance of the difference between groups with MNT addition and the control group (no MNT) is shown (** *p* < 0.01, *** *p* < 0.001, **** *p* < 0.0001).

To study the ability different intracellular concentrations of MNT_R1_ to affect the level of intracellular oxidative processes, we used cDCFH-DA, which is a commonly used fluorescent indicator for this purpose [42]. After entering cells, this dye deacetylates and produces a charged fluorescent product when it interacts with ROS, mainly hydroxyl radicals [43]. To detect them, AML12 cells were incubated with cDCFH-DA for 30 min in Hanks’ solution, after which the cells were washed, and PBS with or without 10 mM hydrogen peroxide was added for 15 min. Flow cytofluorometry has revealed that incubation with hydrogen peroxide under these conditions increases fluorescence in AML12 cells by 2.6 ± 0.2 folds. The average fluorescence of cDCF per cell was not changed after 15 min pre-treatment of AML12 cells with 500 nM MNT_0_, followed by washing with medium (Figure 5a). Similarly, pre-incubation with 500 nM MNT_R1_ for 5 or 10 min did not alter the effect on cDCF fluorescence levels caused by the addition of peroxide after different time points (Figure 5b,c). The pre-incubation of AML12 cells with 500 nM MNT_R1_ for 15 min and subsequent washing with a medium led to a statistically significant decrease in the effect of hydrogen peroxide on cell fluorescence in the range from 2 to 4 h between the addition of MNT_R1_ and peroxide (Figure 5d).

These results demonstrated the possibility of decreasing ROS levels in hepatocyte-derived cells using the inhibitor R1 monobody of the protein–protein Nrf2-Keap1 interaction delivered by MNT_R1_.

### 3.6. MNT with a Detachable R1 Monobody

Earlier, we have shown that the introduction of an FKFL site [33] between the HMP and the monobody to the N-protein of the SARS-CoV-2 virus and cleavage at this site by the endosomal protease cathepsin B led to a significant increase in the affinity of the monobody to the N-protein [22]. Therefore, in order to improve the efficiency of MNT_R1_, a new MNT_clR1_ was obtained in which an FKFL site was introduced between the HMP and the R1 monobody. Using thermophoresis, the dissociation constant of the MNT_clR1_ and tKeap1 complex was determined to be 4.9 ± 1.3 nM (Figure S5). If MNT_clR1_ was cleaved using activated cathepsin B protease, the corresponding dissociation constant became 18 times less, 0.27 ± 0.05 nM (Figure S6), which coincides with the dissociation constant of a free R1 monobody equal to 0.3 nM [27]. Thus, the detachment of the R1 monobody from MNT_R1_ increases its affinity for Keap1 by more than an order of magnitude.

Using the CETSA, melting curves of Nrf2 were obtained in AML12 cells incubated for different times with 500 nM MNT_clR1_ and subsequent washing with Versene solution. From these curves, the dependence of the fraction of active Nrf2, F, at 37 °C on the incubation time of MNT_clR1_ with cells was obtained (Figure 4d, red curve). It is evident that MNT_clR1_ has a significantly greater influence on this fraction than MNT_R1_.

The pre-incubation of AML12 cells with 500 nM MNT_clR1_ for 5 min and subsequent washing with a medium led to a significant decrease in the effect of hydrogen peroxide on cell fluorescence in the range from 2 to 5 h between the addition of MNT_R1_ and peroxide (Figure 5e). Whereas, under the same conditions, the incubation of cells with MNT_R1_ did not have a noticeable effect (Figure 5b).

The obtained data show that the use of MNTs with a detachable R1 monobody significantly increases the effectiveness of MNTs in protecting cells from the effects of hydrogen peroxide.

## 4. Discussion

The current approach to impacting regulatory intracellular proteins can be divided mainly into two directions: the use of small molecules and the use of macromolecules [15,44]. Small molecules are effective as protein–protein interaction inhibitors on targets with pockets and small surface areas. However, this approach is hardly suitable for a significant fraction of intracellular proteins, many of which have large interaction surfaces [45]. On the other hand, antibodies or antibody mimetics are able to adapt to almost any protein surface [46]. The main problems with their use as intracellular agents are their poor penetration into the cell and their lack of selectivity for specific cell types [47,48]. This problem can be solved by using cellular systems of macromolecular transport [17]. One of the possible options for such a solution is the development of multifunctional transport systems that combine transport modules recognized by the corresponding cell systems. A promising application of this approach is MNTs, which can enhance the effectiveness of cytotoxic agents by targeting them to the most damage-sensitive compartment within a cell (for a review, see [21]). In the most common version, the MNT contains four main modules. The ligand module is required to identify a specified cell type and receptor- mediated endocytosis into a cell. The endosomolytic module serves for the pH-dependent release of MNT into the cytosol, which prevents degradation in lysosomes and allows further transport to other compartments of the cell. The module for transport into the nucleus contains NLS that interacts with importins for transport through the nuclear pore. The carrier module is used to spatially separate other modules [21]. Using MNTs based on this principle, we have achieved a significant enhancement in the effectiveness of photosensitizers and Auger electron emitters. This has been demonstrated using various MNTs, both in cell cultures and in the treatment of experimental tumors in mice.

EGFR-expressing cells are one of the most frequently used targets for targeted drug delivery [49]. Recently, we developed MNTs to deliver the R1 monobody against Keap1 in cells that express this receptor [23]. Keap1 is attached to the outer membrane of the mitochondria via the PGAM5 protein [50]. Therefore, the insertion of the R1 monobody sequence into the MNT structure resulted in the delivery of a photosensitizer attached to this MNT_R1_ to the mitochondria, enhancing its cytotoxicity when compared to a free photosensitizer or a photosensitizer conjugated to the MNT without R1. We decided to investigate whether it is possible to activate the antioxidant system Nrf2/ARE using the Keap1 binding module. To examine ARE activation, we used a model system that included the ARE-controlled luciferase gene. For these experiments, modules for binding to Keap1 were fused with the C-terminus of the MNT scaffold. A comparison of sequences from Nrf2 containing the ETGE motif, DEETGE, showed that longer sequences were more effective at activating luciferase when placed under the control of AREs (Table 1). The most effective constructs in this model system were those in which a long sequence of Nrf2, as well as the R1 monobody, were fused with the NLS-deficient MNT scaffold. This suggests that transport into the nucleus decreases the effective concentration of the construct competing for Keap1 in the cytoplasm. The construct with the R1 monobody demonstrated greater ARE-dependent activation compared to the one with the Nrf2 sequence; however, this 30% difference was not statistically significant. Both constructs were equipped with a ligand module for binding to EGFR, and the corresponding proteins were produced in the bacterial system. MNT_R1_ had a higher yield and purity than the protein containing the Nrf2-derived sequence; hence, MNT_R1_ was chosen for further investigations.

We previously showed that MNT_R1_ is able to interact with Keap1 in target cells [23]. Based on these initial findings, we decided to investigate how our MNTs influence the Nrf2 levels in the immortalized hepatocyte AML12. The addition of MNT_R1_ led to a rapid increase in the immunofluorescence of Nrf2 both in the cytoplasm and in the nucleus (Figure 1 and Figure 2). This shows that MNT_R1_ competes with Nrf2 for Keap1 binding and reduces Nrf2 degradation. Our data on the increased level of Nrf2 in both the cytoplasm and nucleus when exposed to Nrf2 activators is consistent with the findings of other researchers who have worked with hepatocyte-derived cells [51,52]. MNT_0_ slightly increased the level of Nrf2 in cells, which is not surprising, given its ability to bind to Keap1 [23]. The discrepancy in the effects of the examined MNTs on Nrf2 levels suggests that the action of MNT_R1_ is unrelated to the implications of its binding to EGFR. Apparently, this effect is due to increased competition for binding to Keap1. This should allow the newly synthesized Nrf2 to enter the nucleus and exert its effect [53]. It should be noted that the addition of MNT has a very rapid effect on the level of Nrf2 in AML12 cells (see Section 3.2). Various approaches, including FLIM-FRET and CETSA, have corroborated the suggestion of competitive binding to Keap1 (see Section 3.3 and Section 3.4).

The FLIM-FRET method was utilized to analyze the interaction of MNT_R1_ with Keap1 in cells. The results demonstrated energy transfer between AF568 attached to MNT_R1_ and hrGFP, fused with Keap1, which occurred shortly after MNT_R1_ was added to the cells. The frequency distribution of the τ_m_ of hrGFP in control cells follows two Gaussian curves, with peak maxima at 2195 ± 63 and 2427 ± 2 ps, respectively (Figure 3b). After 15 min of incubation with MNT_R1_, the frequency distribution of τ_m_ is best represented by four Gaussian curves with maxima of 824 ± 26, 1962 ± 53, 2289 ± 34, and 2409.4 ± 1.7 ps (Figure 3c). Similarly, at 1 h of incubation of cells with MNT_R1_, the frequency distribution is also described by four Gaussian curves with maxima of 1160 ± 13, 2088 ± 18, 2408 ± 7, and 2509.6 ± 1.1 ps (Figure 3d). According to the magnitude of the lifetimes, the last two peaks correspond to hrGFP fluorescence in the absence of FRET. The first two peaks indicate the presence of FRET, or MNT_R1_:Keap1 complexes. The FRET efficiency was computed based on the first peak, and the average distance between the donor and acceptor was found to be 5.3 nm. This distance confirms our prior estimates of 5.1 ± 0.8 nm [23]. It should be noted that Keap1 forms a dimer in the cell, which helps to explain why the second peak appears [54]. In other words, the Keap1 dimer binds to MNT_R1_ in parallel, with hrGFP on each Keap1 dimer molecule acting as a donor for AF568 on MNT_R1_. The only difference between these two molecules will be the distances between the donor and acceptor. In this case, the second peak corresponds to the donor–acceptor distance of 7.3 nm. In other words, it is possible that the second peak is responsible for Keap1, which interacts with MNT_R1_ indirectly via another Keap1 molecule in the dimer. The proportion of Keap1 molecules capable of interacting with MNT_R1_ is then calculated without taking into account the second peak. In this scenario, the proportion of Keap1 that interacts with MNT_R1_ is 16.5% after 15 min and 40.7% after an hour of incubation. Considering that Nrf2 binds to the Keap1 dimer and MNT_R1_ preferably binds with one of them, it can be assumed that 15 min after the addition of MNT_R1_, more than 30% of the synthesized Keap1 is involved in interactions with MNT_R1_. After an hour, this percentage can increase up to 80%. The rapid kinetics of the process may be due to the fact that, as is known, EGFR endocytosis can occur quite rapidly, on a minute scale [55,56].

CETSA was used to prove that MNT_R1_ can not only bind to Keap1 but also displace Nrf2 from the complex with Keap1. The release of Nrf2 from the complex with Keap1 leads to a noticeable increase in the thermal stability of Nrf2. Therefore, analysis of the melting curves of Nrf2 allowed us to conclude that incubation of AML12 cells with MNT_R1_ leads to an increase in the proportion of active Nrf2 in the cells. At the initial time of interaction with the cell, the intracellular concentration of MNT_R1_ can be varied both by changing the external concentration of MNT_R1_ and by varying the duration of incubation of MNT_R1_ with cells. In the second case, this can be done with greater precision than in the first case. Indeed, taking into account the interaction constant of MNT_R1_ with EGFR (63 nM), the selected concentration of MNT_R1_ (500 nM) is saturating for this receptor. As a result, variations in extracellular MNT_R1_ concentration will have a small and nonlinear effect on its intracellular concentration. It should be noted that after the incubation with MNT_R1_, a period of about 60 min elapsed before the CETSA. This enabled the uptake of bound MNT_R1_ molecules and their subsequent endosome escape, thereby affecting the proportion of active Nrf2 molecules in the cells. Previously, we proposed an approach that quantitatively describes the intracellular interaction of MNT with the Nrf2:Keap1 complex [32]. This enables us to estimate the concentration of MNT_R1_ in the cytoplasm by the proportion of active Nrf2, which in this simplified model was assumed to be free Nrf2 [32]. This concentration is described by the following expression [32]:

(1) $${[M}_{\mathrm{tot}}]=\left(\frac{F}{1-F}\cdot {[\mathrm{Keap}1}_{\mathrm{tot}}]-K_{d1}-\frac{F\cdot [{\mathrm{Nrf}2}_{\mathrm{tot}0}]}{1-F\cdot \left(1-\frac{k_{2}}{k_{1}}\right)}\right)\cdot \frac{K_{d1}\cdot \frac{1-F}{F}+K_{d2}}{K_{d1}}$$

where [Keap1_tot_]—the total concentration of Keap1 in the cytoplasm (269 nM [32]); [M_tot_]—the total concentration of MNT_R1_ in the cytoplasm; [Nrf2_tot0_]—the total concentration of Nrf2 in the cytoplasm without the addition of molecules capable of interacting with Keap1 (62 nM [32]); *k*_1_—degradation rate constant of bound Nrf2; *k*_2_—degradation rate constant of active Nrf2; K_d1_—the dissociation constant of the Nrf2:Keap1 complex (5 nM [57]); K_d2_—the dissociation constant of the MNT_R1_:Keap1 complex. It was taken into account that releasing Nrf2 from the complex with Keap1 increases the concentration of active Nrf2, since the rate of degradation of active Nrf2 is substantially slower than that of the complex with Keap1. In this case, the change in the total concentration of Nrf2 can be described by the dependence:

(2) $$\frac{\left[{\mathrm{Nrf}2}_{\mathrm{tot}}\right]}{\left[{\mathrm{Nrf}2}_{\mathrm{tot}0}\right]}=\frac{1}{1-F\cdot \left(1-\frac{k_{2}}{k_{1}}\right)}$$

To estimate the *k*_2_/*k*_1_ ratio, we used certain relative amounts of Nrf2 in cells when they were incubated with MNT_R1_ or MNT_0_. The ratio of these amounts will describe the ability of the monobody within MNT_R1_ to release Nrf2 from the complex with Keap1. This ratio at 15 min of incubation of the cells with MNT_R1_ and subsequent washing was equal to 1.75 ± 0.33. At this incubation time, the F value is 0.44. If we assume that *k*_2_/*k*_1_ is negligible, then, according to expression (2), this ratio is 1.79. Therefore, in further estimates, the *k*_2_/*k*_1_ ratio was assumed to be zero. Then, using the obtained values of the fraction F, we can calculate the concentrations of MNT_R1_ according to expression (1). These calculated concentrations turned out to be 249 ± 14 nM, 291 ± 12 nM, and 373 ± 17 nM for cases when AML12 cells were incubated with MNT_R1_ for 5, 10, and 15 min, respectively, and then washed. It should be noted that these concentrations were also evaluated by Western blot with MNT antibodies, and they amounted to 264 ± 30, 286 ± 21, and 420 ± 47 nM for incubation times of 5, 10, and 15 min, respectively. There is a good correspondence between the concentrations obtained by two different approaches (according to the Mann–Whitney test, no significant difference is observed), where in one case antibodies against Nrf2 were used and in the other those against MNT.

Thus, despite the strong simplification of the real intracellular processes, the proposed quantitative description of the interaction of MNT_R1_ with the Nrf2:Keap1 complex in cells gives correct results. In particular, this indicates that the proportions of active Nrf2 in the cell at different incubation times with MNT were determined correctly. Next, we checked what proportion of active Nrf2 leads to the activation of the Nrf2 system, i.e., causes a biological effect. This was verified by evaluating the effect of MNT on the content of ROS in the cell after the addition of hydrogen peroxide.

Active Nrf2 is transported from the cytosol to the nucleus, where it can activate the expression of hundreds of genes via AREs [5]. The rate of production of different antioxidant proteins varies greatly, and their concentrations reach their maximum after a period of several hours to tens or even hundreds of hours, while their half-life is several hours [7,58]. The change in the contribution of different antioxidant proteins can explain the highly nonlinear dependence of ROS on the time between the addition of MNT_R1_ and the measurement of fluorescence (Figure 5d). Until now, it was not clear what cytoplasmic concentration of active Nrf2 leads to the production of antioxidant proteins. This was made possible using the dependence of the proportion of active Nrf2 on the incubation time of cells with MNT_R1_ (Figure 4d). Thus, at F = 0.22 ± 0.02 and 249 ± 14 nM MNT_R1_, no protection against the action of peroxide was observed (Figure 5b). Similarly, at F = 0.31 ± 0.02 and 291 ± 12 nM MNT_R1_, no reliable protection of cells from the action of hydrogen peroxide was observed (Figure 5c). A clear biological effect was observed at F = 0.44 ± 0.02 and 373 ± 17 nM MNT_R1_ (Figure 5d). Thus, there is a threshold value for the proportion of active Nrf2, F, lying in a range from 0.31 to 0.45, which leads to the activation of the Nrf2 system and the protection of cells from the action of peroxide. Using expression (2) and estimated values of F, the critical concentration of active Nrf2 that causes this activation was estimated. It lies in the range of 28–49 nM. At the same time, the minimum concentration of MNT_R1_ in the cytoplasm at which a biological effect will be observed lies in the range of 291–373 nM according to the model and 286–420 nM according to direct Western blot assessment. Therefore, when delivering compounds that compete with Nrf2 for binding to Keap1, the delivery system must be highly efficient to provide a visible biological effect.

We have previously shown that the introduction of a FKFL site [33] for the cleavage of the monobody to the N-protein of the SARS-CoV-2 virus from the MNTs in endosomes leads to a noticeable increase in the affinity of the monobody to the N-protein [22]. In order to increase the efficiency of MNT_R1_, this site was introduced into its composition, and the resulting MNT was designated MNT_clR1_. The cleavage of this site was carried out by the endosomal protease cathepsin B. Presumably, the cleaved MNT_clR1_ will interact with the Keap1 protein predominantly via the cleaved R1 monobody. The equilibrium dissociation constant of the Keap1 complex with MNT decreases from 5.4 ± 0.5 nM for MNT_R1_ to 0.27 ± 0.05 nM for cleaved MNT_clR1_. In the latter case, it corresponds to the known values of the dissociation constant of the Keap1 complex with the R1 monobody (0.3 nM [27]). Using CETSA, the dependence of the proportion of active Nrf2, F, on the incubation time of MNT_clR1_ with AML12 cells was obtained (Figure 4d, red curve). Compared to MNT_R1_, this curve shifted to the region of lower incubation times (lower intracellular concentrations), and the maximum possible level of active Nrf2 became higher.

An increase in the proportion of active Nrf2 at short incubation times with MNT_clR1_ was reflected in the protection of cells from the effects of hydrogen peroxide. Thus, 5 min of pre-incubation of AML12 cells with MNT_R1_ did not cause a noticeable protective effect (Figure 5b), while 5 min of pre-incubation of these cells with MNT_clR1_ resulted in the reliable protection of the cells from the effects of hydrogen peroxide (Figure 5e). Indeed, the threshold value of the proportion of active Nrf2 required to observe a biological effect (~0.4) was achieved after 2 min of the incubation of cells with MNT_clR1_, as opposed to 15 min for MNT_R1_ (Figure 4d).

The method we proposed earlier [32] for assessing the concentration of MNTs using CETSA data determines in this case not just the concentration of MNTs but the concentration of R1 monobodies capable of interacting with Keap1. In other words, for MNT_R1_ the concentration of R1 monobody coincides with the concentration of MNT_R1_, but for MNT_clR1_ this coincidence is not necessary. If some of the MNT molecules remain anchored in the endosomal membrane upon exiting the endosomes, then the concentration of R1 monobody for MNT_clR1_ will be higher than for MNT_R1_ under similar conditions. The concentration of R1 monobody for MNT_clR1_ determined by expression (1) at 2 and 5 min of incubation with cells was 214.9 ± 1.3 and 251 ± 9 nM, respectively. These values do not differ significantly (Mann–Whitney test) from the corresponding values for MNT_R1_—195 ± 10 and 240 ± 21 nM, respectively. Thus, the introduction of a cleavage site for the R1 monobody from MNTs in endosomes leads to an increase in the affinity of this monobody for Keap1, but does not affect the efficiency of monobody exit from endosomes.

Our developed MNT-based system serves as an example of the targeted influence of protein–protein interactions of specified cells. Modulating protein–protein interactions is essential for the creation of novel medications, as was covered in the introduction, but it is still difficult because most antibodies cannot enter cells and small molecules are not selective [59,60]. To achieve precise effects on intracellular protein targets, more complex drug delivery systems, such as nanoparticle-based ones, are being developed. Here, we demonstrate the possible route for implementing this approach based on MNTs, which combines functional modules for achieving the effect.

## 5. Conclusions

This paper presents a modular nanotransporter (MNT) containing an endosomolytic module and two antibody-like molecules: an affibody for interaction with the epidermal growth factor receptor and an anti-Keap1 R1 monobody for interaction with the Keap1 inhibitor protein. This MNT has been shown to be able to interact with Keap1 in the cytosol of target cells, causing an increase in the proportion of active Nrf2 and an overall increase in the concentration of Nrf2, both in the cytoplasm and in the cell nucleus. In turn, in the selected target cells, this leads to a decrease in reactive oxygen species caused by hydrogen peroxide. The introduction of a cleavage site for the R1 monobody from MNTs in endosomes leads to an increase in the affinity of the anti-Keap1 monobody, which in turn causes a noticeable decrease in its intracellular concentration required to observe a protective effect. The new approach to influencing intracellular protein–protein interactions demonstrated on the example of the Keap1-Nrf2 system can be further applied to the treatment of a very wide range of different diseases, directly associated with oxidative stress in cells or indirectly, like viral infections, including that of SARS-CoV-2 [5].

## Figures and Tables

**Table 1:** Effect of Nrf2-activating modules fused with the MNT scaffold on luciferase activity controlled by AREs in HEK293T cells.

No	Source Protein	Sequence	NLS	Level of Nrf2/ARE System Activation, Fold to TurboYFP Transfection ± SE (*n*)
1	Nrf2	LQLDEETGE	+	5.5 ± 1 (7)
2	Nrf2	LQLDEETGEFLPIQ	+	31 ± 7 (9)
3	Nrf2	LQLDEETGEFLPIQPAQHIQ	+	32 ± 5 (15)
4	Nrf2	LQLDEETGEFLPIQPAQHIQ	−	41 ± 8 (8)
5	Monobody R1	Full	+	16 ± 3 (6)
6	Monobody R1	Full	−	54 ± 10 (6)
7	p62	KEVDPSTGELQSLQ	+	3.5 ± 0.8 (2)
8	Prothymosin α	AQNEENGEQEADNEVD	+	2.7 ± 0.4 (2)

The sequences that are necessary for interaction with Keap1 are underlined.

## Data Availability

Data are contained within this article and its Supplementary Materials.

## Supplementary Materials

The following supporting information can be downloaded at https://www.mdpi.com/article/10.3390/pharmaceutics16101345/s1: Figure S1. The dependence of relative fluorescence intensities (fluorescence intensity before the beginning of thermophoresis is assumed to be 100%) at 2.5 s after the start of thermophoresis on the concentration of MNT_R1_ at a constant concentration of tKeap1 (5 nM). Standard errors (SEs) of relative fluorescence intensity are presented (*n* = 20). Figure S2. The changes in the Nrf2 level in AML12 cells following MNT_R1_ or sulforaphane (SFN) addition. An amount of 500 nM MNT_R1_ or 10 μM SFN was added to AML12 cell for two hours. The fixed cells were stained by indirect immunofluorescence. Nrf2 was revealed by immunofluorescence (red); cell nuclei were stained with DAPI (blue). Transmitted light images are shown in gray. Bar—20 µm. Figure S3. Typical example of Western blot with anti-Nrf2 antibodies of Nrf2 in complex with Keap1, after 15 min of incubation of AML12 cells with 500 nM MNT_R1_. The cells were heated for 3 min to temperatures of 37, 40, 42, 43, 44, 46, and 48 °C. The molecular mass markers are indicated. Figure S4. Western blot with anti-MNT antibodies for samples with 10 nM (1), 20 nM (2), and 30 nM (3) MNT_R1_, and for lysates of AML12 cells without the addition of MNT_R1_ (4) and with the addition of 500 nM MNT_R1_ for 15 min (5). The dotted line shows the area in which the intensity of the studied sample was determined. Figure S5. The dependence of relative fluorescence intensities (fluorescence intensity before the beginning of thermophoresis is assumed to be 100%) at 2.5 s after the start of thermophoresis on the concentration of MNT_clR1_ at a constant concentration of tKeap1 (0.12 nM). Standard errors (SEs) of relative fluorescence intensity are presented (*n* = 8–16). Figure S6. The dependence of relative fluorescence intensities (fluorescence intensity before the beginning of thermophoresis is assumed to be 100%) at 2.5 s after the start of thermophoresis on the concentration of cleaved MNT_clR1_ at a constant concentration of tKeap1 (0.12 nM). Standard errors (SEs) of relative fluorescence intensity are presented (*n* = 8–16).

## References

[1] Bellezza I., Giambanco I., Minelli A., Donato R. (2018). Nrf2-Keap1 signaling in oxidative and reductive stress. Biochim. Biophys. Acta Mol. Cell Res..

[2] Hayes J.D., Dinkova-Kostova A.T. (2014). The Nrf2 regulatory network provides an interface between redox and intermediary metabolism. Trends Biochem. Sci..

[3] Yamamoto M., Kensler T.W., Motohashi H. (2018). The KEAP1-NRF2 System: A Thiol-Based Sensor-Effector Apparatus for Maintaining Redox Homeostasis. Physiol. Rev..

[4] Forman H.J., Zhang H. (2021). Targeting oxidative stress in disease: Promise and limitations of antioxidant therapy. Nat. Rev. Drug Discov..

[5] Ulasov A.V., Rosenkranz A.A., Georgiev G.P., Sobolev A.S. (2022). Nrf2/Keap1/ARE signaling: Towards specific regulation. Life Sci..

[6] Crisman E., Duarte P., Dauden E., Cuadrado A., Rodriguez-Franco M.I., Lopez M.G., Leon R. (2023). KEAP1-NRF2 protein-protein interaction inhibitors: Design, pharmacological properties and therapeutic potential. Med. Res. Rev..

[7] Dinkova-Kostova A.T., Copple I.M. (2023). Advances and challenges in therapeutic targeting of NRF2. Trends Pharmacol. Sci..

[8] Robledinos-Anton N., Fernandez-Gines R., Manda G., Cuadrado A. (2019). Activators and Inhibitors of NRF2: A Review of Their Potential for Clinical Development. Oxid. Med. Cell Longev..

[9] Suzuki T., Motohashi H., Yamamoto M. (2013). Toward clinical application of the Keap1-Nrf2 pathway. Trends Pharmacol. Sci..

[10] Itoh K., Tong K.I., Yamamoto M. (2004). Molecular mechanism activating Nrf2-Keap1 pathway in regulation of adaptive response to electrophiles. Free Radic. Biol. Med..

[11] Saidu N.E.B., Kavian N., Leroy K., Jacob C., Nicco C., Batteux F., Alexandre J. (2019). Dimethyl fumarate, a two-edged drug: Current status and future directions. Med. Res. Rev..

[12] Vinogradova E.V., Zhang X., Remillard D., Lazar D.C., Suciu R.M., Wang Y., Bianco G., Yamashita Y., Crowley V.M., Schafroth M.A. (2020). An Activity-Guided Map of Electrophile-Cysteine Interactions in Primary Human T Cells. Cell.

[13] Lee S., Hu L. (2020). Nrf2 activation through the inhibition of Keap1-Nrf2 protein-protein interaction. Med. Chem. Res..

[14] Mou Y., Wen S., Li Y.X., Gao X.X., Zhang X., Jiang Z.Y. (2020). Recent progress in Keap1-Nrf2 protein-protein interaction inhibitors. Eur. J. Med. Chem..

[15] Lu H., Zhou Q., He J., Jiang Z., Peng C., Tong R., Shi J. (2020). Recent advances in the development of protein-protein interactions modulators: Mechanisms and clinical trials. Signal Transduct. Target Ther..

[16] Mabonga L., Kappo A.P. (2019). Protein-protein interaction modulators: Advances, successes and remaining challenges. Biophys. Rev..

[17] Rosenkranz A.A., Slastnikova T.A. (2023). Prospects of Using Protein Engineering for Selective Drug Delivery into a Specific Compartment of Target Cells. Pharmaceutics.

[18] Ngo V., Duennwald M.L. (2022). Nrf2 and Oxidative Stress: A General Overview of Mechanisms and Implications in Human Disease. Antioxidants.

[19] Taguchi K., Kensler T.W. (2020). Nrf2 in liver toxicology. Arch. Pharm. Res..

[20] Lee W.M. (2017). Acetaminophen (APAP) hepatotoxicity-Isn’t it time for APAP to go away?. J. Hepatol..

[21] Sobolev A.S. (2018). Modular Nanotransporters for Nuclear-Targeted Delivery of Auger Electron Emitters. Front Pharmacol..

[22] Khramtsov Y.V., Ulasov A.V., Lupanova T.N., Slastnikova T.A., Rosenkranz A.A., Bunin E.S., Georgiev G.P., Sobolev A.S. (2023). Intracellular Degradation of SARS-CoV-2 N-Protein Caused by Modular Nanotransporters Containing Anti-N-Protein Monobody and a Sequence That Recruits the Keap1 E3 Ligase. Pharmaceutics.

[23] Khramtsov Y.V., Ulasov A.V., Slastnikova T.A., Rosenkranz A.A., Lupanova T.N., Georgiev G.P., Sobolev A.S. (2023). Modular Nanotransporters Delivering Biologically Active Molecules to the Surface of Mitochondria. Pharmaceutics.

[24] Ichimura Y., Waguri S., Sou Y.S., Kageyama S., Hasegawa J., Ishimura R., Saito T., Yang Y., Kouno T., Fukutomi T. (2013). Phosphorylation of p62 activates the Keap1-Nrf2 pathway during selective autophagy. Mol. Cell.

[25] Karapetian R.N., Evstafieva A.G., Abaeva I.S., Chichkova N.V., Filonov G.S., Rubtsov Y.P., Sukhacheva E.A., Melnikov S.V., Schneider U., Wanker E.E. (2005). Nuclear oncoprotein prothymosin alpha is a partner of Keap1: Implications for expression of oxidative stress-protecting genes. Mol. Cell Biol..

[26] Chen W., Sun Z., Wang X.J., Jiang T., Huang Z., Fang D., Zhang D.D. (2009). Direct interaction between Nrf2 and p21(Cip1/WAF1) upregulates the Nrf2-mediated antioxidant response. Mol. Cell.

[27] Guntas G., Lewis S.M., Mulvaney K.M., Cloer E.W., Tripathy A., Lane T.R., Major M.B., Kuhlman B. (2016). Engineering a genetically encoded competitive inhibitor of the KEAP1-NRF2 interaction via structure-based design and phage display. Protein Eng. Des. Sel..

[28] Ulasov A.V., Khramtsov Y.V., Trusov G.A., Rosenkranz A.A., Sverdlov E.D., Sobolev A.S. (2011). Properties of PEI-based polyplex nanoparticles that correlate with their transfection efficacy. Mol. Ther..

[29] Grune T., Kehm R., Hohn A., Jung T. (2018). “Cyt/Nuc,” a Customizable and Documenting ImageJ Macro for Evaluation of Protein Distributions Between Cytosol and Nucleus. Biotechnol. J..

[30] Jafari R., Almqvist H., Axelsson H., Ignatushchenko M., Lundback T., Nordlund P., Martinez M.D. (2014). The cellular thermal shift assay for evaluating drug target interactions in cells. Nat. Protoc..

[31] Martinez M.D., Jafari R., Ignatushchenko M., Seki T., Larsson E.A., Dan C., Sreekumar L., Cao Y., Nordlund P. (2013). Monitoring drug target engagement in cells and tissues using the cellular thermal shift assay. Science.

[32] Khramtsov Y.V., Ulasov A.V., Rosenkranz A.A., Slastnikova T.A., Lupanova T.N., Georgiev G.P., Sobolev A.S. (2023). An Approach to Evaluate the Effective Cytoplasmic Concentration of Bioactive Agents Interacting with a Selected Intracellular Target Protein. Pharmaceutics.

[33] Kern H.B., Srinivasan S., Convertine A.J., Hockenbery D., Press O.W., Stayton P.S. (2017). Enzyme-Cleavable Polymeric Micelles for the Intracellular Delivery of Proapoptotic Peptides. Mol. Pharm..

[34] Becker W. (2012). Fluorescence lifetime imaging--techniques and applications. J. Microsc..

[35] Datta R., Heaster T.M., Sharick J.T., Gillette A.A., Skala M.C. (2020). Fluorescence lifetime imaging microscopy: Fundamentals and advances in instrumentation, analysis, and applications. J. Biomed. Opt..

[36] McMahon M., Itoh K., Yamamoto M., Hayes J.D. (2003). Keap1-dependent proteasomal degradation of transcription factor Nrf2 contributes to the negative regulation of antioxidant response element-driven gene expression. J. Biol. Chem..

[37] Taguchi K., Motohashi H., Yamamoto M. (2011). Molecular mechanisms of the Keap1-Nrf2 pathway in stress response and cancer evolution. Genes Cells.

[38] Tong K.I., Padmanabhan B., Kobayashi A., Shang C., Hirotsu Y., Yokoyama S., Yamamoto M. (2007). Different electrostatic potentials define ETGE and DLG motifs as hinge and latch in oxidative stress response. Mol. Cell Biol..

[39] Zhao J., Redell J.B., Moore A.N., Dash P.K. (2011). A novel strategy to activate cytoprotective genes in the injured brain. Biochem. Biophys. Res. Commun..

[40] Iso T., Suzuki T., Baird L., Yamamoto M. (2016). Absolute Amounts and Status of the Nrf2-Keap1-Cul3 Complex within Cells. Mol. Cell Biol..

[41] Komatsu M., Kurokawa H., Waguri S., Taguchi K., Kobayashi A., Ichimura Y., Sou Y.S., Ueno I., Sakamoto A., Tong K.I. (2010). The selective autophagy substrate p62 activates the stress responsive transcription factor Nrf2 through inactivation of Keap1. Nat. Cell Biol..

[42] Murphy M.P., Bayir H., Belousov V., Chang C.J., Davies K.J.A., Davies M.J., Dick T.P., Finkel T., Forman H.J., Janssen-Heininger Y. (2022). Guidelines for measuring reactive oxygen species and oxidative damage in cells and in vivo. Nat. Metab..

[43] Wrona M., Patel K., Wardman P. (2005). Reactivity of 2′,7′-dichlorodihydrofluorescein and dihydrorhodamine 123 and their oxidized forms toward carbonate, nitrogen dioxide, and hydroxyl radicals. Free Radic. Biol. Med..

[44] Chen T., Hong L., Yudistyra V., Vincoff S., Chatterjee P. (2023). Generative design of therapeutics that bind and modulate protein states. Curr. Opin. Biomed. Eng..

[45] Lo C.L., Chothia C., Janin J. (1999). The atomic structure of protein-protein recognition sites. J. Mol. Biol..

[46] Sela-Culang I., Kunik V., Ofran Y. (2013). The structural basis of antibody-antigen recognition. Front. Immunol..

[47] Qian L., Lin X., Gao X., Khan R.U., Liao J.Y., Du S., Ge J., Zeng S., Yao S.Q. (2023). The Dawn of a New Era: Targeting the “Undruggables” with Antibody-Based Therapeutics. Chem. Rev..

[48] Slastnikova T.A., Ulasov A.V., Rosenkranz A.A., Sobolev A.S. (2018). Targeted Intracellular Delivery of Antibodies: The State of the Art. Front. Pharmacol..

[49] Rosenkranz A.A., Slastnikova T.A. (2020). Epidermal Growth Factor Receptor: Key to Selective Intracellular Delivery. Biochemistry.

[50] Lo S.C., Hannink M. (2008). PGAM5 tethers a ternary complex containing Keap1 and Nrf2 to mitochondria. Exp. Cell Res..

[51] Lou Y., Guo Z., Zhu Y., Zhang G., Wang Y., Qi X., Lu L., Liu Z., Wu J. (2019). Astragali radix and its main bioactive compounds activate the Nrf2-mediated signaling pathway to induce P-glycoprotein and breast cancer resistance protein. J. Ethnopharmacol..

[52] Sharath Babu G.R., Anand T., Ilaiyaraja N., Khanum F., Gopalan N. (2017). Pelargonidin Modulates Keap1/Nrf2 Pathway Gene Expression and Ameliorates Citrinin-Induced Oxidative Stress in HepG2 Cells. Front. Pharmacol..

[53] Kobayashi A., Kang M.I., Watai Y., Tong K.I., Shibata T., Uchida K., Yamamoto M. (2006). Oxidative and electrophilic stresses activate Nrf2 through inhibition of ubiquitination activity of Keap1. Mol. Cell Biol..

[54] Ogura T., Tong K.I., Mio K., Maruyama Y., Kurokawa H., Sato C., Yamamoto M. (2010). Keap1 is a forked-stem dimer structure with two large spheres enclosing the intervening, double glycine repeat, and C-terminal domains. Proc. Natl. Acad. Sci. USA.

[55] Orth J.D., Krueger E.W., Weller S.G., McNiven M.A. (2006). A novel endocytic mechanism of epidermal growth factor receptor sequestration and internalization. Cancer Res..

[56] Perez V.M., Zhang T., Paulo J.A., Gygi S., Watkins S.C., Sakurai H., Sorkin A. (2021). Mechanism of p38 MAPK-induced EGFR endocytosis and its crosstalk with ligand-induced pathways. J. Cell Biol..

[57] Tong K.I., Katoh Y., Kusunoki H., Itoh K., Tanaka T., Yamamoto M. (2006). Keap1 recruits Neh2 through binding to ETGE and DLG motifs: Characterization of the two-site molecular recognition model. Mol. Cell Biol..

[58] Knatko E.V., Ibbotson S.H., Zhang Y., Higgins M., Fahey J.W., Talalay P., Dawe R.S., Ferguson J., Huang J.T.-J., Clarke R. (2015). Nrf2 activation protects against solar-simulated ultraviolet radiation in mice and humans. Cancer Prev. Res..

[59] Scott D.E., Bayly A.R., Abell C., Skidmore J. (2016). Small molecules, big targets: Drug discovery faces the protein-protein interaction challenge. Nat. Rev. Drug Discov..

[60] Xie X., Yu T., Li X., Zhang N., Foster L.J., Peng C., Huang W., He G. (2023). Recent advances in targeting the “undruggable” proteins: From drug discovery to clinical trials. Signal Transduct. Target Ther..

