# Supplementary material for: Modular Nanotransporters Deliver Anti-Keap1 Monobody into Mouse Hepatocytes, Thereby Inhibiting Production of Reactive Oxygen Species
Source: Pharmaceutics. 2024 Oct 21;16(10):1345. doi: 10.3390/pharmaceutics16101345 (PMC11511107; doi:10.3390/pharmaceutics16101345)
Supplement: Supplementary file 1 [file pharmaceutics-16-01345-s001.zip › pharmaceutics-3219766-supplementary.pdf]

Supplementary data

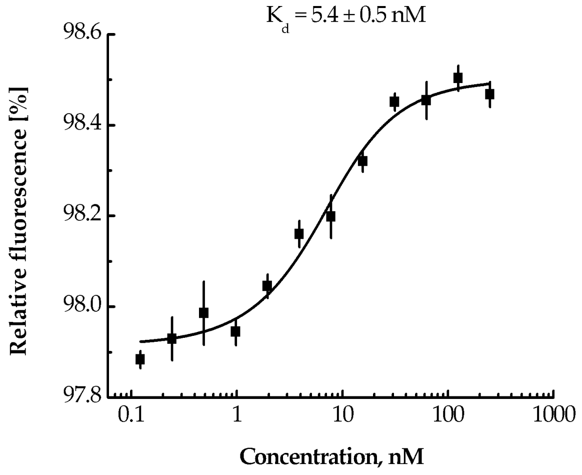

Figure S1. The dependence of relative fluorescence intensities (fluorescence intensity before the beginning of thermophoresis is assumed to be 100%) at 2.5 s after the start of thermophoresis on the concentration of MNT$_{R1}$ at a constant concentration of tKeap1 (5 nM). Standard errors (SE) of relative fluorescence intensity are presented (n = 20).

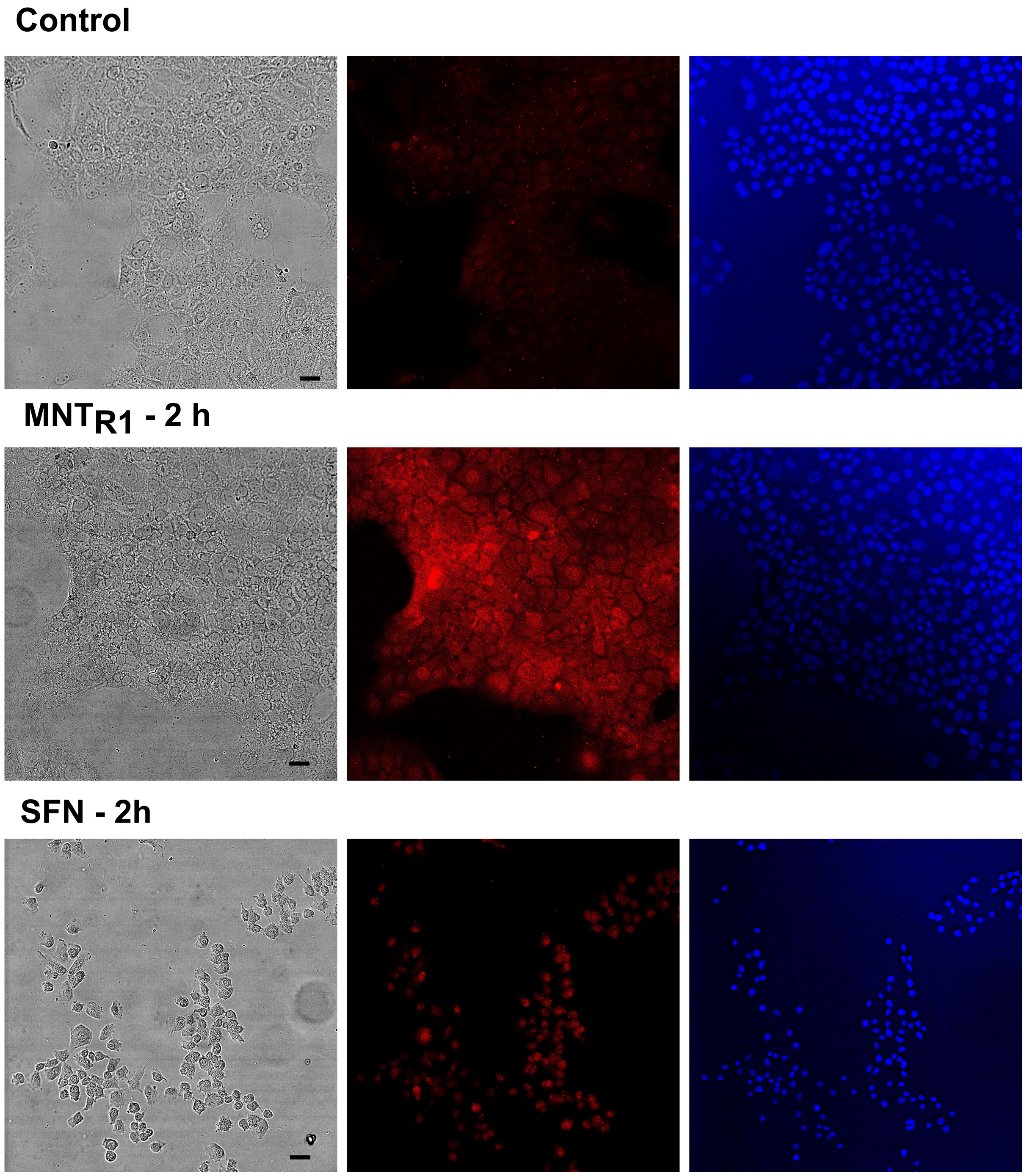

Figure S2. The changes in the Nrf2 level in AML12 cells following MNT$_{R1}$ or sulforaphane (SFN) addition. 500 nM MNT$_{R1}$ or 10 μM SFN were added to AML12 cell for two hours. The fixed cells were stained by indirect immunofluorescence. Nrf2 was revealed by immunofluorescence (red), cell nuclei were stained with DAPI (blue). Transmitted light images are shown in grey. Bar – 20 μm.

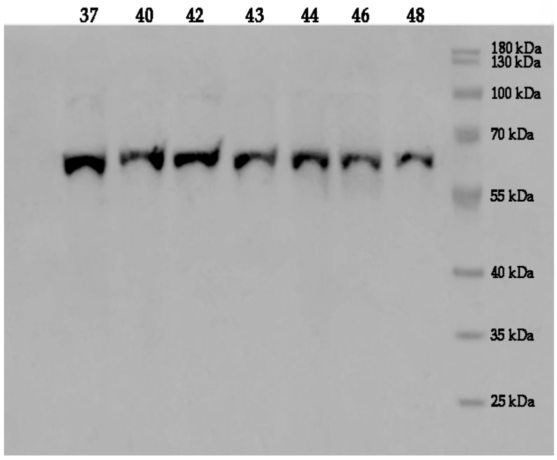

Figure S3. Typical example of Western blot with anti-Nrf2 antibodies of Nrf2 in complex with Keap1, after 15 minutes of incubation of AML12 cells with 500 nM MNT$_{R1}$. The cells were heated for 3 minutes to temperatures of 37, 40, 42, 43, 44, 46 and 48°C. The molecular mass markers are indicated.

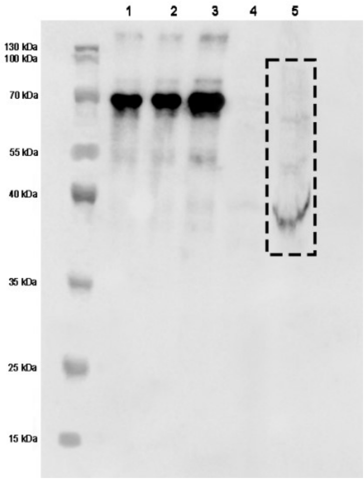

Figure S4. Western blot with anti-MNT antibodies for samples with 10 nM (1), 20 nM (2), and 30 nM (3) MNT$_{R1}$, and for lysates of AML12 cells without the addition of MNT$_{R1}$ (4) and with the addition of 500 nM MNT$_{R1}$ for 15 minutes (5). The dotted line shows the area in which the intensity of the studied sample was determined.

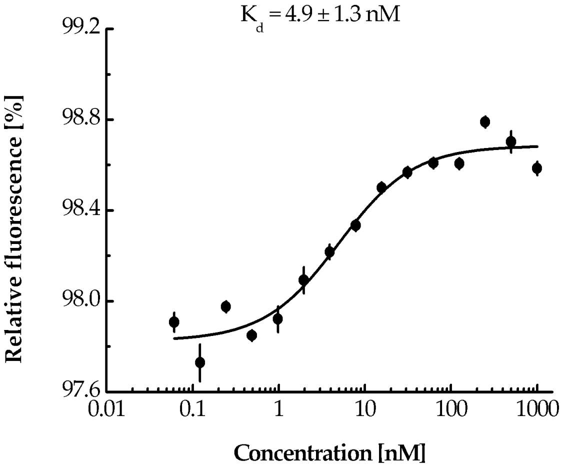

Figure S5. The dependence of relative fluorescence intensities (fluorescence intensity before the beginning of thermophoresis is assumed to be 100%) at 2.5 s after the start of thermophoresis on the concentration of MNT$_{clR1}$ at a constant concentration of tKeap1 (0.12 nM). Standard errors (SE) of relative fluorescence intensity are presented (n = 8–16).

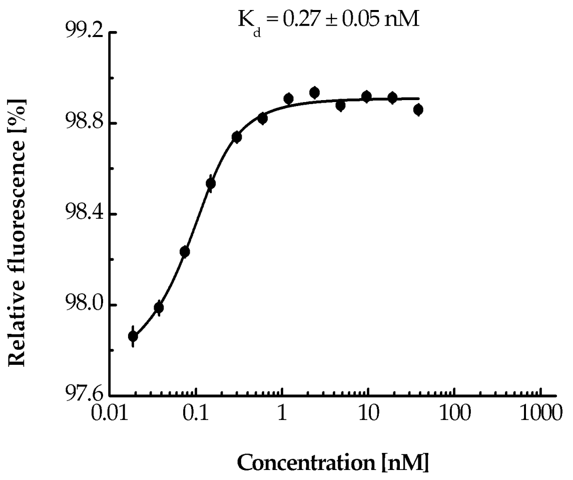

Figure S6. The dependence of relative fluorescence intensities (fluorescence intensity before the beginning of thermophoresis is assumed to be 100%) at 2.5 s after the start of thermophoresis on the concentration of cleaved MNT_{clR1} at a constant concentration of tKeap1 (0.12 nM). Standard errors (SE) of relative fluorescence intensity are presented (*n* = 8–16).
